# SELF-ASSESSED ORAL HEALTH IN DETROIT SENIOR CENTERS

**DOI:** 10.1093/geroni/igad104.2781

**Published:** 2023-12-21

**Authors:** Divesh Byrappagari, Judith Jones

**Affiliations:** University of Detroit Mercy School of Dentistry, Detroit, Michigan, United States; University of Detroit Mercy School of Dentistry, Detroit, Michigan, United States

## Abstract

The objective of the Detroit Mercy Senior Oral Health Equity Project (SOHEP) survey was to gather information from seniors about their opinion regarding their oral health status and satisfaction with their past dental care experience. The questionnaire was developed based on previously tested questions from other national senior oral health surveys. Data were collected using Qualtrics© and analyzed using SPSS©. The majority of the seniors screened were African American (88.9%), female (79.1%) and between the ages of 65 and 85 yrs. Over half (58.2%) reported their oral as Fair or Poor. Over one third (37.3%) reported avoiding eating certain foods due to poor oral health, and 43.3% have not been to a dentist in over 2 years. Although most participants indicated that they have insurance and have good experiences with their past dental care, a significant number of seniors have not used dental care. A community-based program that is accessible to seniors, especially in places where they congregate frequently might be helpful and improve access to preventive dental care for this population.

